# Microglia Mediated Neuroinflammation in Parkinson’s Disease

**DOI:** 10.3390/cells12071012

**Published:** 2023-03-25

**Authors:** Sevim Isik, Bercem Yeman Kiyak, Rumeysa Akbayir, Rama Seyhali, Tahire Arpaci

**Affiliations:** 1 Department of Molecular Biology and Genetics, Faculty of Engineering and Natural Sciences, Uskudar University, Uskudar, Istanbul 34662, Turkey; 2 Stem Cell Research and Application Center (USKOKMER), Uskudar University, Uskudar, Istanbul 34662, Turkey; 3 Department of Molecular Medicine, Institute of Hamidiye Health Sciences, University of Health Sciences, Uskudar, Istanbul 34668, Turkey; 4 Department of Molecular Biology, Institute of Science, Uskudar University, Uskudar, Istanbul 34662, Turkey

**Keywords:** Parkinson’s Disease, neuroinflammation, microglial activation, α-synuclein, M1 phenotype, M2 phenotype, anti-inflammatory phenotype

## Abstract

Parkinson’s Disease (PD) is the second most common neurodegenerative disorder seen, especially in the elderly. Tremor, shaking, movement problems, and difficulty with balance and coordination are among the hallmarks, and dopaminergic neuronal loss in substantia nigra pars compacta of the brain and aggregation of intracellular protein α-synuclein are the pathological characterizations. Neuroinflammation has emerged as an involving mechanism at the initiation and development of PD. It is a complex network of interactions comprising immune and non-immune cells in addition to mediators of the immune response. Microglia, the resident macrophages in the CNS, take on the leading role in regulating neuroinflammation and maintaining homeostasis. Under normal physiological conditions, they exist as “homeostatic” but upon pathological stimuli, they switch to the “reactive state”. Pro-inflammatory (M1) and anti-inflammatory (M2) phenotypes are used to classify microglial activity with each phenotype having its own markers and released mediators. When M1 microglia are persistent, they will contribute to various inflammatory diseases, including neurodegenerative diseases, such as PD. In this review, we focus on the role of microglia mediated neuroinflammation in PD and also signaling pathways, receptors, and mediators involved in the process, presenting the studies that associate microglia-mediated inflammation with PD. A better understanding of this complex network and interactions is important in seeking new therapies for PD and possibly other neurodegenerative diseases.

## 1. Introduction

Parkinson’s disease (PD) is the most common form of movement disorder in the elderly and its prevalence ranges from 0.5 to 1% in adults aged 60–69 and 1–3% in those over 80 years [1,2]. The typical symptoms of PD are tremor, movement problems, and balance and coordination difficulties. The pathological feature is loss of dopaminergic neurons in the substantia nigra pars compacta (SNpc) in the basal ganglia of the brain and extensive aggregation of intracellular protein α-synuclein (α-syn) [3].

In addition to the SNpc, there is neuronal loss in the cerebral cortex, basal ganglia, thalamus, locus coeruleus, and brain stem, especially the dorsal motor nucleus [4,5]. Different brain regions are affected depending on the stage of PD. Initially, loss of dopaminergic neurons takes place only in the ventrolateral SN, while other dopaminergic neurons are preserved in the midbrain [6,7]. However, this limited neuronal degeneration spreads to larger areas in the late stage of PD. On the other hand, Lewy bodies (LB), the other major neuropathology [8] mostly composed of accumulated α-syn, first appear in cholinergic and monoaminergic brainstem neurons as well as in neurons of the olfactory pathway. However, as the disease progresses, they also appear in limbic and neocortical brain areas [9]. Both depletion of dopaminergic neurons in SNpc [10,11] and α-syn aggregations in neurons are not specific neuropathologies of PD. However, when these two major pathologies occur together, they are particular indicators of the crucial diagnosis of idiopathic PD.

It has been demonstrated that the development of PD can be influenced by a number of molecular pathways. When proteins produced by PD-associated genes are impaired, these mechanisms—including genes, α-syn proteostasis, neurotransmitters, autophagy, mitochondrial dysfunction, and oxidative stress—can lead to neuropathology comparable to sporadic PD [12]. Although age is an important risk factor in PD pathogenesis, the etiology of PD is still unclear. However, familial forms of PD, which comprises <10% of PD cases, have been linked to mutations occurring in PD-associated genes, such as SNCA (PARK1), LRRK2 (PARK8), PRKN (PARK2), PINK1 (PARK6), and DJ-1 (PARK7) [13,14]. Mutations in these genes result in autosomal recessive and autosomal dominant monogenic forms of familial PD [15]. According to large genome-wide association studies (GWAS), some of the PD-associated genes are also involved in sporadic PD [16]. Moreover, the sporadic form of PD has been shown to have several variations in these genes, including SNCA, LRRK2, PINK1, GBA, and PRKN, which encode α-syn, leucine rich repeat kinase 2, PTEN-induced kinase 1 (PINK1), glucocerebrosidase, also known as lysosomal acid glucosylceramidase, and E3 ubiquitin-protein ligase parkin, respectively. In addition, these genes encode proteins capable of regulating the immune response [15].

The factors that led to the disease’s development are not yet fully understood in the remaining 90% of the cases (idiopathic PD). Increased expression of wild-type α-syn is observed in idiopathic PD, although the most common mutations in familial PD are not in the SNCA gene [17,18]. Inclusions resulting from abnormal deposition of intracellular α-syn protein are a common pathology in PD patients [19]. Point mutations occurring in SNCA gene or duplication of the entire locus result in the autosomal dominant type of PD [20]. In addition, GWAS have identified a single-nucleotide polymorphism (SNP) associated with the SNCA gene that causes enhanced α-syn expression [16]. These results support the idea that α-syn is an important factor contributing to the neuropathophysiology of PD. 

Impaired autophagy, which may cause aggregation of α-syn [21], has been associated with neurodegenerative diseases, including PD [22]. Molecular mechanisms related to autophagy, inflammation, or oxidative stress may reinforce each other in the progression of neurodegenerative diseases. In destroyed neurons in PD, mitochondrial dysfunction and α-syn aggregation are observed together. These pathological changes are thought to exacerbate each other [12]. Studies have implicated that mitochondrial dysfunction results in oxidative stress, which has a key role in cell death caused by various pathophysiological conditions. When reactive oxygen species (ROS) generation in a cell exceeds the accessibility of antioxidants or radical scavengers, this leads to oxidative stress. Mitochondria contribute strongly to ROS production [23]. It has been shown that oxidative stress increases in the PD-patients’ brain tissues. However, it is not known exactly at which stages of the disease oxidative stress occurs. Increased oxidative stress and mitochondrial dysfunction are related to genes [24] and can lead to the destruction of lysosomes [25] providing further evidence that a variety of putative pathogenetic pathways are interconnected in PD [26,27]. Autophagy induced by oxidative stress may also lead to the degeneration of SNpc neurons [28]. Persistent inflammation, mediated mainly by microglial cells, may lead to oxidative stress and mitochondrial dysfunction resulting in aggregation of α-syn and contributing to neuropathological mechanisms that end up with dopaminergic neuronal damage in PD [29].

In recent years, interest in neuroinflammation as an involved mechanism in PD pathology has increased. With age, homeostasis of the brain is damaged because the activation of survival mechanisms decreases. As a result, neuroinflammation may develop in PD. Neuroinflammation is an essential immune response in the central nervous system (CNS) to maintain neuronal health. On the other hand, its neurotoxicity may exacerbate the neuronal injury [30,31]. However, it is not known whether neuroinflammation is a cause or consequence of PD. Therefore, further studies are needed to clarify the role of neuroinflammation in the mechanisms underlying PD pathology.

In this review, we focus on microglia and their functions in the inflammatory response and discuss the contribution of microglia-mediated inflammation to PD with scientific studies.

## 2. Neuroinflammation and PD

When inflammation occurs in the CNS, it is called neuroinflammation. It occurs in response to a variety of inflammatory signals, such as trauma, infection, aging, genetic risk factors, and pathogenic protein aggregates. In response to these stimuli, responses from microglia, neurons, astrocytes, and peripheral immune cells work in hand with cytokines, chemokines, and the complement system to organize an inflammatory response. This response may directly result in oxidative stress that is critical for neuron survival. The brain’s resident immune cells, microglia and astrocytes, play a critical role in this response. Microglia are able to undergo physiological changes to become active, known as the “reactive state”, and take on their role in secreting inflammatory molecules. It all comes to the point whether the inflammatory stimuli are acute or chronic. Acute inflammation leads to prompt repair of tissue following environmental factors, such as infection, toxins, or traumatic injury. Chronic neuroinflammatory responses, on the other hand, are usually associated with neurodegenerative diseases [32]. Circumstances, such as α-syn misfolding [33], mitochondrial dysfunction [23], and polymorphisms in immune-related genes [34], are some of the cases responsible for chronic neuroinflammation. In the case of PD, as a matter of fact, numerous changes occur inside the dopaminergic neurons in the SNpc. These changes include α-syn release, protein degradation problems, mitochondrial dysfunction, etc. As a result, the surrounding environment is subjected to an inflammatory response, which in turn attracts other cell types [35].

In the “reactive state”, microglia are able to perform what is known as synaptic pruning while astrocytes produce factors that cause the release of complement components by neurons. The increased production and release of complement proteins then leads to phagocytosis of neurons by microglia [36]. At this point, neurodegeneration begins to demonstrate its pathophysiological features. Microglia in the reactive state produce high levels of pro-inflammatory cytokines and chemokines eventually leading to a disrupted blood brain barrier (BBB). The disrupted BBB becomes vulnerable to the leak of peripheral immune cells (B cells, T cells, and monocytes), which enhance cytokine release by glial cells. Along with glial cells, neurons are also capable of contributing to the release of inflammatory mediators [37].

The fine balance throughout the neuroinflammatory process is maintained by glial cells. In order to ensure the homeostasis in the brain following any type of neurological insult, astrocytes increase their rate of proliferation to form the “glial scar” forming a barrier for the nervous tissue to prevent spreading of the injury. Both microglia and astrocytes then release specific factors aiding tissue regeneration starting with extracellular matrix (ECM) reconstructions, neovascularization, and angiogenesis. In doing so, they work with the peripheral immune cells to create a safe environment for the remaining neurons, to support immigration of stem cells into the area to replace dead neurons, and ensure remodeling of axons and neuron repair [38,39].

### 2.1. Role of Innate and Adaptive Immunity and Their Members

PD should not be considered a disease concerning only the brain but rather a multi-systemic disease that encompasses peripheral immune cells involving monocytes and T cells of the innate and adaptive immune systems, respectively [35].

Acute and chronic responses are usually differentiated depending on the involvement of cell types of innate and adaptive immunity. When triggering factors cause dysfunctional neurons and/or activated microglia, they influence each other. Microglia then activate T cells by antigen presentation and subsequently B cells to produce antibodies. When this goes on as a continuous cycle, the adaptive immune system eventually develops a chronic inflammatory response and damages neurons in PD [40].

In the case of a neurological insult, the cells of the immune system work together to generate a response. Glial cells in the CNS immediately produce signals, such as cytokines, chemokines, growth factors, and other metabolites, to bring the myeloid cells of the peripheral system into action. This leads to a broader inflammatory response as other immune cells, the T lymphocytes, become involved in the process. Neurons also contribute to the activation of immune cells by releasing inflammatory signals [41,42].

As a major player in neuroinflammation, reactive state microglia are also responsible for the activation of astrocytes. Among the many properties of astrocytes is the maintenance of BBB integrity and permeability. They contribute to neuron plasticity, survival, development, protection, and tissue restoration by secreting neurotrophic factors: glial cell line-derived neurotrophic factor (GDNF), brain-derived neurotrophic factor (BDNF), nerve growth factor (NGF), and cerebral dopamine neurotrophic factor (CDNF). These factors fight ROS through the production of antioxidants and, as already mentioned, form the glial scar for the regeneration of nervous tissue [39,43]. Working together, microglia and astrocytes can also eliminate extracellular α-syn exerting a protective effect on neurons [44,45]. Just like microglia, astrocytes also have pro- and anti-inflammatory properties. In the case of an inflammatory state, they can regulate the activation of microglia [46]. As for T lymphocytes, they have been found to be increased in SN of PD patients [47]. Moreover, studies have shown that humoral adaptive immunity is also involved in the process of PD pathogenicity [48]. Taken together, these results indicate that PD involves activation of both innate and adaptive immune systems.

### 2.2. Role of Inflammatory Mediators

It is well known that when there is a defect in the CNS or the peripheral nervous system (PNS), pro-inflammatory cytokines are known to increase and cause inflammation leading to neurotoxicity. The role of these cytokines cannot be underestimated as they are considered important promoters of neurodegeneration. The levels of pro-inflammatory cytokines (IL-2, IL-10, IL-4, IL-6, tumor necrosis factor α (TNFα), and interferon γ (IFNγ)) have been found to increase in the serum of PD patients. They have proposed two mechanisms by which this elevation occurs. First, they found that number of lymphocytes increased in concert with increased cytokine levels, so the lymphocytes could be responsible for releasing more cytokines. Alternatively, these cytokines were increased in the brain in the first place and may directly leak into the bloodstream through the BBB [49].

Glial cells, astrocytes, and microglia respond as a team to neuroinflammation. Both release the cytokines mentioned above as well as ROS and chemokines through the activation of TLRs [38]. Moreover, cytokine release from microglia (especially IL-1α, TNFα, and C1q) triggers the activation of astrocytes to upregulate this response further by becoming neurotoxic themselves, thereby setting forth a synergistic effect [46]. Glial cells indeed work in two alternative pathways: pro- and anti-inflammatory. Simply, when microglia secrete pro-inflammatory mediators (TNFα, IL-6, IL-1β, IL-18, IL-23, IFNγ, nitric oxide (NO), and ROS), they stimulate astrocytes to result in neuron and oligodendrocyte death. When microglia secrete anti-inflammatory (IL-4, IL-10, transforming growth factor β (TGF-β), insulin growth factor 1 (IGF-1), vascular endothelial growth factor (VEGF), BDNF, and platelet-derived growth factor (PDGF)) mediators, they lead to damage repair [38].

### 2.3. Role of Neurons

Neurons are not only influenced by microglia but are also effectors of microglia controlling their functions. Dysfunctional SN dopaminergic neurons can trigger inflammation in PD. Structural changes in α-syn can act as a damage-associated molecular pattern (DAMP). Different forms of defective α-syn (monomeric, oligomeric, or fibrillar) are able to recognize membrane receptors in microglia causing their activation and the release of pro-inflammatory cytokines and ROS. In most cases, this interaction results in an inflammation with subsequent elimination of the defective protein. α-syn is thus not only the pathological feature of the disease but also the driving force of inflammation. However, there are studies suggesting that its monomeric forms increase the phagocytic capacity of microglia, whereas aggregated forms do the opposite. Inhibition of phagocytosis contributes to further neurodegeneration [35,50].

### 2.4. Role of Neuroinflammation-Triggering Factors

PD is not a disease of the brain that affects only one system, but its pathogenicity is exacerbated when genetic, environmental, and age-related factors are added. Many studies have suggested a relationship between PD and the stress factors to be explained in the following.

Aging plays a significant role in promoting neurodegeneration. Oxidative stress and mitochondrial dysfunction are two important factors to consider in the aging process. In addition to inflammatory signals, mitochondrial respiration also generates ROS, which increases with age. Under normal conditions, ROS is deliberately kept at a low level to regulate certain signaling pathways of physiological processes. Increased ROS in turn results in increased DNA damage and genomic instability. Increased oxidative stress as a result of increased ROS puts mitochondria, its major producer, at risk. This risk may lead to dysfunctional mitochondria and consequent adverse effects on cell survival [23,51,52]. Although low levels of α-syn are found in mitochondria, its accumulation will interfere with the organelle function [53,54]. As a matter of fact, aside from physiological aging, immune system aging needs a great deal of consideration. While inflammation is usually beneficial at young ages under normal physiological conditions, it becomes unfavorable with age. The term inflammaging was introduced by Franceschi et al. at this point, where there is a persistent burden of inflammatory factors in the human body. The optimal conditions for a balance between pro- and anti-inflammatory mediators cannot be maintained leading to chronic diseases, such as neurodegeneration [55].

PD—the causative and immune-related genes play an important role in the etiology of PD. According to the meta-analysis performed with a GWAS of PD, there were more than 90 risk variants that helped to explain the heritability of the disease (16–36%) [56]. There were some immune-associated genes involved in the regulation of immune system function: Bone Marrow Stromal Cell Antigen 1 (BST1) involved in neutrophil adhesion and migration; synaptotagmin 11 (SYT11) involved in secretion of cytokines and microglial phagocytosis; Transmembrane Protein 175 (TMEM175), which is involved in the regulation of lysosomal pH and lysosomal catalytic activity; and Granulin Precursor (GRN), which is involved in cytokine-like activity and cell growth, proliferation, and tumorigenicity [15].

Additionally, some of the PD-causing genes are also involved in the regulation of immune-system related events. The main feature of PD, α-syn, is directly responsible for microglial activation by acting as damage-associated molecular patterns (DAMPs) [57]. Both its aggregation and overexpression both lead to the activation of microglia resulting in increased pro-inflammatory mediators and eventually dopaminergic neuron degeneration [58]. Silencing of α-syn resulted in similar results as its overexpression. It caused degeneration in rat SNpc and caused neuroinflammation and death of neurons in the region [59].

In addition to SNCA, the other genes involved in the etiology of PD also have a role in neuroinflammation. LRRK2 is known to be highly expressed in immune cells, suggesting that inflammation and regulation of immune responses are among the many roles of this gene. PD patients who have mutations of this gene have shown high inflammatory cytokine levels in their serum [60] and high LRRK2 expression in their B cells, T cells, and monocytes [61]. PRKN and PINK1 are responsible for the maintenance of mitochondrial homeostasis and are also necessary for proper immune regulation. Their mutations impair mitochondrial function and lead to ROS generation along with an inflammatory response [62]. PD patients with and without GBA mutations were examined for their biochemical profiles and the results showed that the patients with the mutation had higher levels of inflammatory mediators in their serum samples [63].

The Environment and the factors that are associated with it should not be overlooked in the development of diseases. Factors include, but are not limited to, infections [64,65,66,67], diet, exposure to pesticides [68,69,70], usage of caffeine and tobacco, altered microbiota [71,72,73] activate pathways that lead to neuroinflammation, mitochondrial dysfunction, excessive oxidative damage, impaired autophagy, and protein aggregation. Indeed, genetic and environmental parameters play a common role in the pathogenicity of PD activating similar pathways [74].

Glial cells in the CNS cooperate with immune cells in the PNS to carry out the complicated and active process of neuroinflammation. Since they are the most numerous immune cells in the CNS, microglia have the most control when it comes to neurodegeneration resulting from pathological insults. Back in 1988, the extent of the microglial response was uncovered as a typical histopathological feature of PD [75]. There are numerous studies demonstrating the significance of these cells and their contribution to PD. In the following sections, we will discuss the role that microglia-mediated neuroinflammation plays in PD.

## 3. Microglia

### 3.1. Origin and Characteristics of Microglia

The resident macrophages of the CNS, the microglia, are derived from a single mesodermal lineage, whereas the other brain parenchymal cells are derived from several neuroectodermal lineages. They mostly arise from primitive macrophages of the yolk sac [76]. At an early stage of development, a number of mesoblastic cells infiltrate the nervous system and differentiate into neuroglia. They are almost evenly distributed throughout the CNS of the mature brain with minimal variation [77]. Microglia make up between 0.5% and 16.6% of all brain cells depending on the anatomical region [78].

Microglia express a variety of membrane receptors for chemokines, cytokines, neurotransmitters, hormones, and purines to respond immediately to alterations in homeostasis of the brain. The fractalkine CX3C-chemokine receptor 1 (CX3CR1), colony stimulating factor 1 receptor (Csf-1R), integrin CD11b, surface glycoproteins F4/80 and CD68, ionized calcium binding adaptor molecule 1 (IBA1), and pan-hematopoietic CD45 are highly expressed markers in steady-state microglia that are also common to other tissue-resident macrophages [79,80]. Several genes have been identified that distinguish microglia from other cells in the CNS and PNS and are known as “signature factors” of microglia. These are TMEM119, Olfml3, P2ry12, Tgfbr1, Fcrls, Hexb, and Siglec-H. However, it is not yet certain whether they are involved in defining the movement, shape, and function of microglia [81]. TGF is also crucial for modulating microglial survival and phenotypic differentiation as demonstrated by a significantly decreased microglial density in mice lacking the TGF receptor [82].

### 3.2. Functions of Microglia

**Figure 1:**
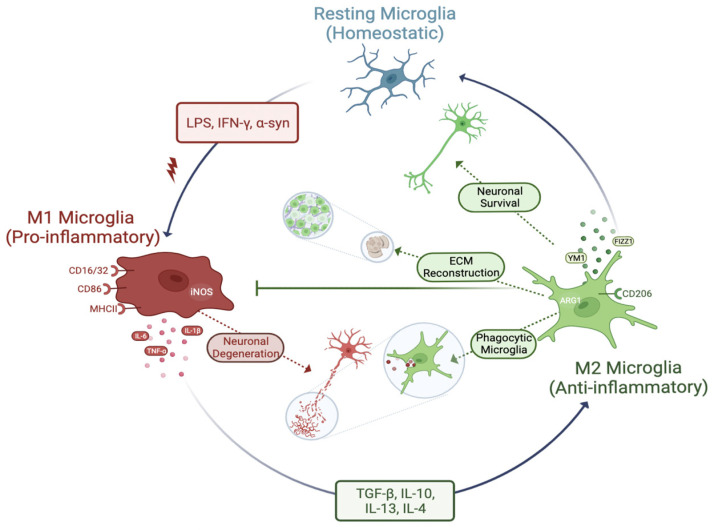
M1 and M2 microglia phenotypes. Under physiological circumstances, microglia exhibit the homeostatic microglia phenotype. Depending on the environment in which they are reactive and the factors in which they are stimulated, they can change into “pro-inflammatory (M1)” or “anti-inflammatory (M2)” phenotypes. Microglia play important roles in maintaining the health of neurons, including pruning and remodeling synapses, controlling myelination, and removing pathological proteins that are misfolded through neurogenesis and phagocytosis. Microglia are also responsible for maintaining the homeostasis of brain tissue. Additionally, depending on the type of activation, microglia secrete numerous trophic factors, cytokines, and chemokines to aid in neuronal survival. Pathogenic molecules, such as LPS and/or IFN, or protein aggregates, such as α-syn, stimulate microglia into the pro-inflammatory phenotype, which then releases inflammatory molecules, such as ROS and other pro-inflammatory cytokines, including IL-1β, iNOS, TNFα, and others. Persistent exposure of microglia to these inflammatory mediators may result in neuronal damage. Contrarily, mediators, such as TGF-β, IL-4, IL-10, and IL-13, induce the M1 to M2 transition. The M2 phenotype of microglia contribute to the processes in phagocytosis, ECM rebuilding, and neuronal survival by secreting such factors as Ym1 and FIZZ1.

Microglia play a critical role in maintaining homeostasis and host defense against pathogens and CNS diseases under both physiological and pathological conditions. Microglia have both neuroprotective and neurotoxic effects suggesting that their biological importance in the CNS is multidimensional. Normal functions of microglia are critical at all stages of development, from embryonic development to maturation and aging [83,84]. Microglia govern neurogenesis throughout CNS development through the apoptosis-coupled phagocytosis of newborn cells in the subgranular zone [85] and promote neuron survival by congregating around axons and releasing trophic factors, such as IGF-1 [86]. Furthermore, throughout postnatal development, microglia actively carry out synaptic pruning actively for normal brain development by eliminating redundant synaptic connections through phagocytosis [87]. In a healthy adult brain, microglia also have crucial functions in neuronal plasticity in the brain. In vivo imaging and high-resolution electron microscopy investigations reveal that tiny processes of microglia actively interact with dendritic spines suggesting that microglia are actively involved in remodeling synaptic circuits [88]. Bidirectional neuron–microglia communication is particularly important for neuronal circuits and brain connections. It is generally accepted that communication between microglial receptors and factors secreted by neurons maintains the surveillance state of microglia [89]. Microglia also contribute to myelination by phagocytizing excess oligodendrocytes and improperly formed myelin membranes. Furthermore, microglia promote oligodendrocyte survival and differentiation and control oligodendrocyte apoptosis by secreting a variety of trophic factors, chemokines, and cytokines [90,91]. Through their processes, microglia constantly survey their microenvironment to perform their housekeeping functions, such as cleaning up cellular debris, synaptic pruning and remodeling, and regulation of myelination (Figure 1). These processes are sensitive to any kind of changes in the physiology of their environment. While maintaining CNS homeostasis, they are able to search for and detect immunological threats. Therefore, microglia have a very flexible morphology and can undergo morphological changes in response to environmental demand [87].

### 3.3. Phenotypes of Microglia (Activation of Microglia)

Under physiologically normal conditions, microglia are known to be in a *homeostatic* or *ramified state* with extensive contacts with other cells (neurons, oligodendrocytes, and astrocytes) and also with blood vessels. They constantly surf around to seek out any kind of change and sense pathology in their immediate environment [92]. Once the neurological system has been compromised and upon pathological stimuli, they switch to a *reactive state*. Pro-inflammatory (M1) and anti-inflammatory (M2) phenotypic features are frequently used to express the microglial activity. When activated, microglia move to the site of damage or pathogen and mediate the immune response like a double-edged sword to either heal or worsen the situation. Keeping microglia under control requires complex regulation during their transition from the homeostatic state to the reactive state [93] (Figure 1). Even though only two categories are commonly used, several types of activation states and various targets and receptors are thought to be continuously present to regulate the microglial response. There is a spectrum of functional states for microglia that form a continuum rather than discrete phenotypes. Importantly, in most cases, their phenotype is transient rather than definitive. These phenotypes are merely temporary states of microglia. Unlike homeostatic microglia, microglia in the reactive state generally have an amoeboid shape, short processes, expanded soma, and de novo cell surface receptor expression and release both pro-inflammatory and anti-inflammatory factors that can enhance or slow disease progression [94]. In the healthy state, immune responses are fine regulated during either their onset or their resolution to keep tissue homeostasis. On the contrary, in the pathologic state, immune responses are uncontrolled and prone to be excessively immune imbalanced, which is closely associated with cell loss or dysfunction in inflammatory conditions [95].

Cytokine traffic between blood and brain, triggered by infection, immune system substances produced by BBB endothelial cells, and immune signals in the periphery released by the autonomic nervous system, all influence microglia in a continuous manner. Aside from the responses of microglia to their immediate environment, the physiological function of the CNS immune system is dependent entirely on the intricate but poorly understood interactions between the PNS and CNS [41,42].

#### 3.3.1. M1 Phenotype

The M1 phenotype is used to define the state of classical activation. The term “M1 microglia” also refers to microglia in this state. Classical activation represents the body’s first line of defense and is manifested by the pro-inflammatory and killing-promoting properties of microglia. Microglia secrete pro-inflammatory cytokines (IL-1β, IL-6, IL-12, IL-17, IL-18, IL-23, TNFα, and IFNγ) and NO, and chemokines, such as CC-chemokine ligand 2 (CCL2), are polarized to M1 phenotype [96,97]. Additionally, microglia exhibit phenotypic markers after M1 activation, such as major histocompatibility complex class II (MHC II), inducible nitric oxide synthase (iNOS), CD86, cyclooxygenase-2 (COX2), and additional molecules, such as ROS, prostaglandin E2 (PGE2), and reactive nitrogen species (RNS). These coordinated actions are thought to eliminate foreign pathogens and other threats and induce T cells to trigger an adaptive immune response [93].

The existence of the neurotoxic M1 phenotype of microglia in the presence of neurodegeneration indicates either a replacement of the M1 phenotype by an M2 phenotype or a transition from the M2 to M1 phenotype during the course of the disease [98]. α-syn aggregates, amyloid beta plaques, and other unidentified stimuli may induce a particular microglial M1 phenotypic expression. Microglia stimulated by these activators are triggered by the MAPK-associated protein-1 (MAPK/AP-1) and inhibitor of kappa B/NF-κB (IκB/NF-κB) signaling cascade and stimulate pro-inflammatory cytokines [99,100,101].

##### ***Potent Activators of M1 Phenotype of Microglia:*** 

α-syn aggregation or inability to remove it from the cell leads to its secretion, which can cause α-syn accumulation in or out of the neurons ultimately resulting in neuronal toxicity. Ongoing research is investigating how microglia respond when exposed to either an excessive amount or a mutant form of α-syn. The clearance of an excessive amount of α-syn from the cytoplasm is facilitated mainly by activated microglia. It has also been observed that extracellular α-syn (monomers, oligomers, and fibrils) regulate the inflammatory response of microglia. They operate as DAMPs and activate receptors, such as TLR2 and other receptors (e.g., CD11b), of innate immune responses on the microglia cell surface [35,57]. Extracellular α-syn induces the NF-κB signaling pathway in microglia, which is an important component of the microglial response to inflammation. This signaling pathway led to priming and activation of the NLR family pyrin domain containing 3 (NLRP3) inflammasome, ultimately promoting microglial IL-1β release. This results in mitochondrial dysfunction followed by pro-inflammatory cytokine production, which in turn damages dopaminergic neurons via the NF-κB pathway [102,103]. In addition, abnormal α-syn is known to induce an excessive microglial activation while adversely affecting synaptic function and mitochondrial homeostasis [35]. Aggregated α-syn released from neurons causes dysregulation of the microglial immune response by blocking the phagocytosis of microglia resulting in impaired removal of accumulated proteins or cellular debris, which in turn contributes to neurodegeneration as seen in PD [104].

LPS is the most prevalent and well-known inflammatory stimulus for microglia. It has been demonstrated that LPS causes dopaminergic neuron death in vivo and in vitro by inducing M1 microglia via TLRs [105]. LPS is normally activated by binding to TLR4 and TLR2 receptors on the microglial membrane. It is then attached to myeloid differentiation protein 2 (MD2) (TLR4/MD2), with the involvement of the co-receptors CD14 and LPS-binding protein (LBP). Activation of TLR4 by LPS triggers downstream signaling molecules, such as myeloid differentiation primary response protein 88 (MyD88) and toll/IL-1 receptor (TIR) domain-containing adaptor inducing interferon β (TRIF), as well as transcription factors, such as NF-κB, signal transducer and activator of transcription 5 (STAT5), and interferon regulatory factors (IRFs) [106]. This sets off a chain reaction that leads to the upregulation of M1-associated gene transcription, such as chemokines, cytokines, and genes expressed in pro-inflammatory responses. 

IFNγ or type II interferon, is a cytokine that performs an essential function in both innate and adaptive immunity against infections by viruses, some bacteria, and protozoa. Both activation of macrophages/microglia and induction of the production of MHC II molecules are significant functions of IFNγ. It induces the M1 phenotype of microglia via the Janus kinase/signal transducer and activator of transcription (JAK/STAT) signaling pathway. This activation is carried out through the IFNγ receptors 1 and 2 (IFNγR1/2), which in turn activate and phosphorylate JAK1/2. The phosphorylated JAK1/2 activates STAT1, followed by translocation of STAT1 to the nucleus and IRFs. This signaling pathway activates the transcription of genes encoding cytokines, chemokines, and other molecules associated with the M1 inflammatory response [93,107].

Granulocyte-macrophage colony-stimulating factor (GM-CSF) has also been shown to stimulate M1 activation alternatively via the CD11b receptor [108]. On the other hand, unlike LPS and IFNγ, GM-CSF is believed to be capable of inducing pleomorphic activation states, which exhibit aspects of both the M1 and M2 phenotypes [109].

##### ***Toll-like Receptors (TLRs)*** 

###### *TLR4* 

TLRs are crucial for the development of innate immunity. Structural motifs, also known as PAMPs, can activate these receptors resulting in the maturation of antigen presentation cells (APCs) and triggering the pro-inflammatory cytokine production. TLR4, along with a number of other TLRs, are highly expressed in microglia [110]. TLR4 can be triggered by a diverse array of PAMPs, including the bacterial component LPS and proteins of viral envelope [38], heat shock proteins [111], and hyaluronic acid [112]. Indeed, TLR4-dependent activation of microglia, which is responsible for persistent neuroinflammation, is well documented in a variety of neurodegenerative diseases, including PD. According to the findings of the study, the absence of TLR4 inhibits the progression of neuroinflammation associated with PD via regulation of the NF-κB, activator protein 1 (AP-1), and inflammasome pathways suggesting that this receptor has the potential to be an effective therapeutic target in neurodegenerative diseases [113].

###### *TLR2* 

TLR2 receptors are able to recognize a broad spectrum of PAMPs, including those produced by viruses, fungi, bacteria, and parasites, such as components of the bacterial cell wall (LPS and lipopeptides) and protein components of bacteria (flagellin and nucleic acids). In addition, they have the ability to respond to certain DAMPs derived from both endogenous cells and the environment [100]. Misfolded and/or aggregated α-syn initiates a series of processes, including microglial activation, that result in the death of dopaminergic neurons. TLR2 is directly activated by extracellular α-syn released from neurons into the environment [57]. Microglial cells can uptake this extracellular α-syn and this results in the activation of microglia with ROS, inflammatory cytokine release, and ultimately dopaminergic neuronal death [58]. Microglial activation and pro-inflammatory cytokine production are the end consequence of α-syn fibrils activating the TLR2-NFκB pathway in glial cells [114].

##### ***IκB/NF-κB Pathway*** 

The NF-κB transcription factor family is able to modulate multiple signaling pathways in the immune system. It provides a mechanism for cells to react to numerous pro-inflammatory stimuli. Agents, such as bacterial toxins, viruses, UV light, and oxidative stress, can activate the NF-κB pathway. Other agents that can also activate NF-κB include carcinogens, tumor promoters, and various mitogens [115]. NF-κB is associated with the M1 microglial activation. Inhibition of NF-κB p65/p50 subunits in the hypothalamus, which has a significant function in whole body aging, reduces the expression of inflammatory genes, including COX2 and iNOS [116]. NF-κB is a significant factor in a wide range of autoimmune diseases, as well as inflammation. It has been suggested as a novel therapeutic target for inflammatory diseases [117]. NF-κB promotes the M1 microglia phenotype; on the other hand, there are a number of regulators that can inhibit NF-κB to switch microglia from M1 to M2. This indicates that NF-κB could be an important factor in microglia polarization. When in the cytoplasm, it is present in its inactive form without being stimulated to bind to IκB/NF-κB [100,118]. After a series of processes, the IκB protein is degraded and NF-κB p50/p65 is allowed to move into the nucleus. It then binds to the promoters of the genes it targets, such as IL-6, TNFα, and COX2, which are involved in neuroinflammation-mediated pathology and activates their transcription [119]. Depletion of M1 macrophages controls the inflammatory effects and blunts the loss of tyrosine hydroxylase (TH) positive nigral neurons and deficits produced by MPTP. The findings highlight the importance of M1 microglia in PD pathogenesis and suggest their suppression as a promising treatment strategy for neurodegeneration [120].

##### ***MAPK/AP-1 Pathway*** 

MAPK are serine/threonine protein kinases that have significant cellular roles, such as proliferation, differentiation, and apoptosis [121]. The MAPK signaling cascade consists of three main branches, p38 MAPK, c-Jun NH2-terminal kinase (JNK), and extracellular signal-regulated kinase (ERK1/2) [122]. Previous reports show that p38 and JNK are closely associated with the microglial activation, including AP-1-dependent induction of pro-inflammatory factors at the transcriptional level. Stimulation of microglia with LPS can activate nuclear JNK by increasing its amount. Moreover, inhibition of JNK results in a decrease in the metabolic activity induced by LPS and the transcription of AP-1 target genes (such as TNFα, IL-6, COX2, and monocyte chemoattractant protein-1 (MCP-1)) [123]. Inactivation of p38α/MAPK, which is essential for LPS-induced degeneration of neurons, prevents the results of LPS-induced activation of microglia via TNFα signaling [124]. ERK1/2 and JNK kinases also play significant roles in redox signaling of microglia [125]. In vivo data on neurodegenerative diseases also suggest that p38 and JNK MAPKs have a role in the activation of microglia [126]. The increased M1/M2 polarization of microglia results in anti-neuroinflammatory effects via MAPK-dependent inactivation. These data show that the MAPK pathway inactivation is a highly effective strategy for treating neuroinflammatory diseases [127].

#### 3.3.2. M2 Phenotype

“M2 microglia’’ is a term that encompasses both “alternative activation” and “acquired deactivation” states. They are involved in a variety of processes, including immunoregulation, slowing of inflammatory responses, healing, and recovery from injury. Microglia are polarized into the “alternative activation” of the M2 phenotype when anti-inflammatory stimuli (such as IL-4, IL-10, IL-13, TGFβ, and glucocorticoids) are present. This phenotype is associated with smoothing inflammation and restoring homeostasis. Following classical activation, an anti-inflammatory and reparative phase is immediately activated, which aids to reduce inflammation, repair damaged tissue, and regenerate the ECM [128,129]. “Acquired deactivation” of the M2 phenotype is another condition that decreases acute inflammation. It is typically produced by the engulfment of cells undergoing apoptosis or by exposure to TGF-β and IL-10 (anti-inflammatory cytokines). The morphological differences and coexistence of the two phenotypes are not yet known. Despite this, it is possible that the two phenotypes may merge in a variety of settings, leading to the development of pathogenic manifestations of inflammation in neurodegenerative diseases [98]. M2 polarized microglia release anti-inflammatory molecules, such as arginase-1 (Arg1), which aid in wound healing and matrix deposition. These cells also produce growth factors, such as FIZZ1, IGF-1, and chitinase 3-like 3 (Ym1), which facilitate the deposition of ECM. Additionally, M2 polarized microglia are characterized by cell surface M2 phenotype receptors, including the triggering receptor expressed on myeloid cells 2 receptor (TREM2) and the mannose receptor (CD206) [92,130,131].

Microglial activation has a significant impact on the pathophysiology of neuroinflammatory and neurodegenerative diseases, such as AD, PD, and others (amyotrophic lateral sclerosis (ALS), multiple sclerosis, traumatic brain injury, and stroke) [98,132]. On the other hand, the idea of microglia polarization is currently debated because the M1/M2 paradigm may oversimplify in vivo activation. The heterogeneity of the microglia phenotype related to various pathogenic situations can be further defined using transcriptomic and proteomic analyses [133].

There are three subtypes of M2 microglia: M2a, M2b, and M2c. Each have different markers and inducers. The M2a state can be triggered by either IL-4 or IL-13 and is related to phagocytosis and tissue healing. Binding of IL-4 to various receptors causes the transcription of target genes related to the M2a state, such as CD206, suppressor of cytokine signaling 3 (SOCS3), and scavenger receptors (SRs). This state is the primary option for microglial activation. The M2b state is triggered when both TLRs and IL-1 receptors are engaged. It produces pro- and anti-inflammatory cytokines (TNFα, IL-6, and IL-10) and contributes to the recruitment of regulatory T cells to the inflammation site. Activation of M2b leads to the release of IL-10 and cell surface expression of CD86 and MHC II. The M2c activation state is engaged in both anti-inflammatory and healing processes in the body. It is triggered by IL-10 and glucocorticoid hormones and results in the suppression of pro-inflammatory cytokines related to the M1 phenotype. It is also involved in the remodeling of tissue and matrix deposition [99,134,135].

#### 3.3.3. M1 to M2 Phenotype Transition

The shift from the M1 to M2 phenotype (from pro-inflammatory to a regulatory/anti-inflammatory state) contributes to enhanced functional outcomes and homeostatic restoration. When microglia are exposed to certain chemicals, such as IL-10, glatiramer acetate, beta interferons, and others, their phenotype may change from M1 to M2. Despite the obvious differences in function between M1 and M2 microglial phenotypes, it is possible that M1- and M2-related factors are expressed simultaneously or that a mixed M1/M2 phenotype is expressed in an injury environment due to the presence of distinct subpopulations expressing distinct phenotypes [131].

Neurodegenerative disorders are often accompanied by neuroinflammation, and the possibility of stimulating the transition from M1 to M2 by a pharmacological approach could be of therapeutic importance in these cases.

##### ***TREM2 Receptors*** 

TREM2 expression is related to an activated phenotype of a cell that carries out critical protective functions for the maintenance of CNS tissue homeostasis, including reconstruction of the tissue, regulation of inflammation in the immediate environment, and phagocytosis of cells undergoing apoptosis [136]. TREM2 is responsible for controlling actin polymerization as well as the architecture of the cytoskeleton by increasing protein tyrosine kinase ERK and signaling through the adaptor protein DNAX-activating protein 12 (DAP12). In addition, TREM2 stimulates the phagocytosis of microglia via ERK by increasing the expression of C-C motif chemokine receptor 7 (CCR7) on the surface of the cell and stimulating the migration of chemokine-activated cells toward CCR7 ligands [137]. It is possible that inhibition of the TLR pathway by TREM2/DAP12 signaling plays a role in reducing inflammation by inhibiting the production of pro-inflammatory cytokines. TREM2 is involved in NF-κB regulation by its role in reducing inflammation, activating the of PI3K/Akt pathway, and suppressing the TLR signaling by inhibiting MAPK signaling [138]. Additionally, TREM2 enhances the survival of microglia by triggering the Wnt/catenin signaling pathway. Through its interaction with DAP12, TREM2 promotes the PI3K/Akt signaling pathway resulting in the stimulation of anti-apoptotic and pro-mitotic genes [139].

##### ***JAK/STAT Pathway*** 

One of the most important signaling pathways for modulating innate and adaptive immunity is the JAK/STAT pathway. Its three main components are JAKs, STATs, and receptors. To control the expression of certain genes, STAT undergoes phosphorylation, dimerization, and nuclear translocation once JAK binds to its ligand. It is known that the JAK/STAT system is one of the significant pathways that regulates a variety of signals to keep homeostasis in inflammatory circumstances and cellular functions that mediate innate and adaptive immunity allowing the production of cytokines and IFNs [140]. When phosphorylation of the JAK/STAT pathway is blocked, the microglial phenotype switches from a pro-inflammatory to an anti-inflammatory state, and microglia subsequently secrete less inflammatory cytokines [141]. The JAK/STAT pathway can also promote microglia transition into the M2 phenotype, for example, through JAK2/STAT6 [142], where STAT6 controls genes associated with the microglia M2 phenotype, such as Arg1, CD206, Fizz1, Ctl3, and Ym1 [143]. On the other hand, there are also studies demonstrating that STAT1 activity is crucial for M1 macrophage polarization. Activation of the JAK2/STAT1 pathway enhances the macrophage polarization into the M1 phenotype because it increases the production of the genes encoding IL-1β, CXC motif chemokine 10 (CXCL10), and TNFα [144,145]. JAK2/STAT3 activation induces microglia to polarize into the M1 phenotype, which as a result causes inflammatory harm to the hippocampus [146]. Much evidence suggests that the JAK/STAT pathway plays a part in the development of PD by modifying microglial polarization [147]. PD patients have elevated levels of IFNγ and IL-6, two of the most powerful JAK/STAT pathway activators [148,149]. As can be seen, there are studies supporting both the M1 phenotype and the M2 phenotype of the JAK/STAT pathway in microglia. Since the effects of JAK/STATs on microglia are not fully elucidated, further research is required to identify other JAK/STAT pathways with potential effects on the polarization of microglia.

##### ***AMPK-Dependent Pathway*** 

Inflammatory mediators, such as histamine and thrombin, increase intracellular calcium (Ca^2+^) influx during inflammation [150]. When Ca^2+^ binds to calmodulin (CaM), it stimulates the Ca^2+^/CaM kinase cascade activation along with calcium/calmodulin-dependent protein kinase kinase β (CaMKKβ). Once CaMKKβ becomes active, it phosphorylates AMP-activated protein kinase (AMPK) [151]. AMPK functions in maintaining steady cellular energy levels by stimulating glucose and fatty acid uptake and oxidation during periods of energy depletion [152]. It is also involved in the polarization of microglia from M1 to M2 [153]. The microglial transition from the M1 to M2 phenotype via AMPK has been demonstrated in many studies [154,155]. Multiple neurodegenerative diseases with mitochondrial dysfunction and poor energy metabolism have been associated with AMPK dysregulation. The leading cause of PD, aging, is associated with the decline of mitochondrial activity, energy metabolism, and AMPK function [156,157]. Activating PD-relevant AMPK-dependent pathways may be developed into a good strategy to minimize neurodegeneration and eventually limit the course of PD, after extensive research and better tailored pharmacotherapeutics.

##### ***PI3 Kinase/Akt Pathway*** 

The PI3K/Akt is essential in health and disease and regulates cellular functions, such as metabolism, apoptosis, and cell proliferation. The Ser and Thr kinase Akt, also called protein kinase B (PKB), is activated by stimulation of G-protein-coupled receptors (GPCR) or receptor tyrosine kinases (RTK), which then triggers Akt activation by phosphorylating its T308 and S473 residues [158]. The PI3K/Akt pathway and other significant pathways interact with each other. For instance, the PI3K/Akt pathway’s downstream component is NF-κB [159], and in order to activate the PI3K/Akt pathway, phosphorylated JAK is necessary. Akt indirectly inhibits the AMPK signaling pathway. The PI3K/Akt pathway is suppressed by ERK activation, and the ERK pathway is also inhibited by Akt [160]. Activated Akt is crucial to initiate immune responses as dysregulation of PI3K/Akt is seen in the pathophysiology of a number of nervous system diseases [161]. Microglial activity and activation appear to be closely related to PI3K/Akt signaling in the neuroinflammation of the brain [162]. Numerous studies demonstrated that decreased PI3K/Akt signaling leads to an M1 phenotype, whereas its increase promotes the M2 phenotype [163]. Anti-inflammatory modulators of Akt activity result in lower pro-inflammatory microglial activation. These findings suggest that PI3K/Akt signaling in microglia may induce anti-inflammation [164]. On the other hand, LPS-activated microglia in the brain increased the production of pro-inflammatory factors through activation of PI3K/Akt [165]. These findings suggest that the PI3K/Akt pathway may also result in neuroinflammation by activating microglia.

##### ***Rho/ROCK Pathway*** 

Rho, which belongs to the Ras superfamily of small GTPases, is an important member of the small Rho GTPases. The downstream effector protein of the small Rho GTPase, Rho-kinase (ROCK), a serine/threonine kinase, is demonstrated to be involved in numerous physiological processes in the cell, such as movement, morphology, polarity, contraction, gene expression, and cell division [166]. Its dysregulation can be a causative factor in the pathophysiology of various neurodegenerative diseases [167]. The Rho/ROCK signaling pathway is also an essential component of the process of inflammation [168]. ROCK has a potential regulatory influence on the microglia phenotype. Microglia can switch from an M1 to an M2 state in response to the ROCK inhibitor Fasudil, which reduces ROCK2 activity, thereby decreasing pro-inflammatory molecules, including NO, IL-1β, IL-6, and TNFα, while raising the anti-inflammatory IL-10. In addition, Fasudil reduces the microglial inflammatory response and/or induces microglial anti-inflammatory polarization, preventing the negative consequences of excessive neuroinflammation by blocking the NF-κB pathway [169]. Moreover, ROCK has a regulatory effect on the mitochondrial ROS. In a study, activation of ROCK led to mitochondrial dysfunction, while inhibition of ROCK activity with a ROCK inhibitor (Y-27632) led to recovery of mitochondrial function and a decrease in the level of ROS [170]. The findings show that inhibiting ROCK activity improves mitochondrial abnormalities and effective treatment techniques may be implemented to contribute to mitochondrial dysfunction throughout aging and aging-related diseases. Additionally, the effects of anti-inflammation and blocking the Rho/ROCK system may be attributable to their ability to promote microglia polarization toward the M2 phenotype. This will facilitate the development of novel treatments for neurodegenerative diseases. Thus, neurodegenerative diseases, including PD, may benefit from treatment approaches that modulate microglial activity by inhibiting ROCK [171].

##### ***Notch Pathway*** 

**Figure 2:**
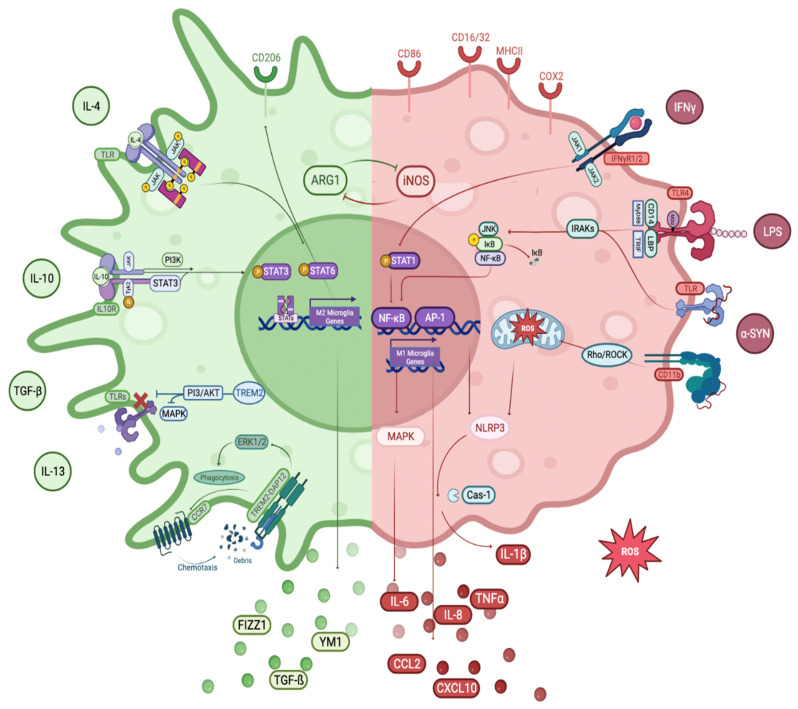
Overview of microglial M1 and M2 signaling pathways in PD. The right side of the figure represents the M1 microglial phenotype and its related signaling pathways. LPS, which is one of the main M1 microglia activators, binds to MD2-bound TLR4 on the cell surface via LBP (LPS-binding protein) and CD14, which acts as a co-receptor. The resulting complex binds to TRIF and Myd88 by interacting with the cytoplasmic domain of TLRs and individual TIR domains. Activated TLR phosphorylates the IKK complex, which consists of MAP kinases, such as IKKβ, JNK, through autophosphorylation of IRAKs, thereby inducing translocation and activation of transcription factors NF-κB and AP-1 and initiates upregulation of M1-associated gene transcription. M1 activation by IFNγ occurs by initiation of the signaling pathway by IFNγR1/2, phosphorylation of STAT1 and interferon regulatory factors, and translocation to the nucleus via JAK1/2. Aggregates of α-syn released into the extracellular space bind to TLR2 following the working mechanism of TLR2s, triggering activation of NF-kB and subsequent NF-kB-dependent upregulation of NLRP3 and production of proinflammatory cytokines. NLRP3 activation provides caspase-1-mediated release of IL-1ß and IL-8. In addition, α-syn clusters are recognized by CD11b and induce mitochondrial ROS generation by impairing mitochondrial function via the Rho/ROCK pathway. In addition, the activation of the M1 phenotype contributes to the regulation of intracellular iNOS, cell surface markers (CD86, CD16/32, and MHC II), M1-related pro-inflammatory cytokines (IL-1β, IL-6, IL-12, IL-17, IL-18, IL-23, and TNFα), and chemokines (CCL2, CXCL10). The left side of the figure represents the M2 microglial phenotype and its related signaling pathways. M2 status is mainly induced by anti-inflammatory stimuli, such as IL-4, IL-10, IL-13, TGF-β, and glucocorticoids. IL-4 binds to IL-4R causing phosphorylation of JAKs/STAT6 and translocation of STAT6 to the nucleus. Activated STAT6 specifically leads to the transcription of M2-related genes, including intracellular components, such as CD206 and cytokine signal suppressor 3 (SOCS3). IL-10 binds to IL-10R1/2, enabling STAT3 to be phosphorylated and translocated to the nucleus via the JAK/STAT signal cascade and PI3K. STAT3 translocation inhibits M1-related proinflammatory cytokines and upregulation of IL-10 and TGF-β. TGF-β increases ARG1 expression and decreases iNOS and COX2. M2 microglia activation releases anti-inflammatory molecules, such as Arg-1, IGF-1, Ym1, and FIZZ1, which contribute to matrix deposition and wound healing. With the increase of the M2 phenotype, TREM2 inhibits the PI3/AKT signaling pathway, TLRs, and MAPK and provides the transition to the M2 phenotype and the inhibition of the M1 phenotype. In addition, the TREM2/DAP12 complex stimulates ERK1/2 by regulating actin polymerization and cytoskeleton with ERK1/2 activation. This activation increases the expression of CCR7 on the cell surface and provides chemotactic migration towards CCR7 ligands. In addition, TREM2 activation stimulates microglial phagocytosis in the same way. Activation of the PI3K/Akt pathway by TREM2/DAP12 contributes to the regulation of NF-κB and inhibition of TLR signaling by blocking MAPK signaling.

Notch is a widespread signaling pathway that controls the fate of the cell. There are four Notch receptors in mice and humans, as well as five Delta/Serrate/Lag-2 ligands (DSL) [172]. These ligands are transmembrane proteins that facilitate contact-dependent interactions among the cells. Activated Notch receptors are cleaved by γ-secretase releasing the Notch intracellular domain (NICD) from the membrane [173]. The NICD moves to the nucleus to facilitate gene transcription by acting as a co-activator [174]. The Notch signaling pathway controls various cellular functions [175] as well as the microglial polarization [176]. Inhibition of the Notch1 signaling pathway reversed LPS-induced M1 microglia polarization, which resulted in increased M2 microglia polarization and decreased inflammatory cytokine production [177]. These results indicate that inhibition of Notch signaling contributes greatly to the transition of the M1 phenotype of microglia to the M2 phenotype (Figure 2).

## 4. Evidence of Microglia-Mediated Neuroinflammation in PD

Microglial activation is often associated with neurodegeneration, a degenerative process that underlies the end pathology of neurodegenerative diseases. Many studies have shown that microglial activation is indeed associated with PD pathology.

### 4.1. Human Studies

Pro-inflammatory microglia have been demonstrated in numerous studies in postmortem and patient PD brains and extracellular fluids obtained from PD patients. 

#### 4.1.1. Postmortem Studies

An amoeboid morphology and MHC II positive microglia are features of M1 phenotype microglia. DAMPs generated by dying neurons or pro-inflammatory mediators, misfolded and/or aggregated proteins, such as α-syn, and signals sent by TLRs promote the acquisition of pro-inflammatory microglia that tend to increase MHC I and II expression. The first neuropathological evidence for the role of microglia in PD was reported in 1988. In that study, researchers found that post-mortem SN samples from PD patients had increased numbers of reactive microglia compared with age-matched healthy controls. Reactive microglia are characterized by the activation of MHC II proteins involved in antigen presentation and inflammatory signaling. Although these MHC II positive cells had a wide range of microglial morphologies, they were highly enriched in reactive ameboid shapes suggesting an association with neuropathological activity [75]. This finding was later confirmed by other studies denoting that increasing numbers of MHC II positive microglia caused the progression of neuronal degeneration in SN. Moreover, in addition to SN and the putamen, the number of MHC II positive microglia was considerably higher in the hippocampus, transentorhinal cortex, cingulate cortex, and temporal cortex in PD compared to healthy controls [178].

The presence of the aggregated form of α-syn in the postmortem PD brain was shown to correlate with the activation of microglia in that brain [179]. However, autopsy of the brains of patients diagnosed with PD, particularly autopsy of the SNpc and basal ganglia, showed that the density of nitrated α-syn (N-α-syn) is increased where microglia are activated. Also, analysis of NF-κB expression revealed that expression of NF-κB in the SNpc is increased, especially when stimulated by N-α-syn, explaining the involvement of NF-κB in the inflammatory response of PD [180].

High throughput single-cell RNA sequencing (scRNAseq) is used to understand cellular heterogeneity at the transcriptional level. The snRNAseq analysis was applied in a postmortem study of the human PD brain, where frozen postmortem midbrain tissue from idiopathic PD patients showed a disease-specific increase in nigral microglia compared to healthy controls, and the observed amoeboid shape of microglia indicates their reactive state [181]. Microglial signaling pathways involved in PD progression, such as phagocytosis and release of inflammatory factors, are more prominent in humans than in animal models [182].

Infiltration of CD4+ and CD8+ T cells in the SN was demonstrated by the immunohistochemical staining of the PD postmortem brain, and the increase in their density in the SN was also significantly observed [47].

#### 4.1.2. Patient Studies

##### Brain PET Imaging Studies

PET imaging has been used to study microglial activation in vivo to better understand its involvement in the pathogenesis of PD. The first translocator protein (TSPO), known to be overexpressed on the mitochondrial membrane of reactive microglia, PET tracer was developed to be used mainly for the imaging of neuroinflammation [183,184].

In a recent study, the existence and distribution of neuroinflammatory responses were investigated using [^18^F]-DPA714 in PD patients. Corresponding imaging biomarkers with clinical status and DAT imaging were evaluated. All participants were genotyped for the TSPO polymorphism and scanned with [^18^F]-DPA714 and [^11^C]-PE2I. In comparison with control subjects, PD patients had higher [^18^F]-DPA714 non-displaceable binding potential (BP_ND_) in the putamen, midbrain, and frontal cortical areas. Activated microglia were observed bilaterally in the midbrain but were noticeably more apparent on the side most affected by the disease. However, there was no relation between disease severity and binding to TSPO in the midbrain [185].

Another recent study investigated the activation of microglial cells in the brain and their connection with adaptive immunity-related peripheral inflammatory biomarkers, disease severity, and function of dopaminergic presynaptic neurons. They used ^18^F-PBR06 (TSPO marker indicating microglial activity) PET/MR to evaluate microglial activation and ^18^F-FP-DTBZ for dopaminergic denervation. In a comparison between early-stage PD patients and control subjects, PD patients had a higher ^18^F-PBR06-PET standardized uptake value ratio (SUVR) in the putamen, especially on the side ipsilateral to the motor onset. In the brainstem, ^18^F-PBR06-PET SUVR was favorably correlated with ^18^F-FP-DTBZ-PET SUVR [186].

##### Extracellular Biological Fluid Studies

Inflammatory molecules in cerebrospinal fluid (CSF) and blood can be used as potential biomarkers to determine the neuroinflammatory pathogenesis of PD.

CSF is a suitable source that can be exploited to identify molecular alterations underlying the pathogenesis of PD, since it is difficult to get live human neurons from PD patients. For example, the level of oligomeric α-syn in the CSF of PD patients and neurological controls, diagnosed with either hydrocephalus, vascular parkinsonism, or drug-induced parkinsonism, resulted in elevated oligomeric α-syn in PD patients compared to control [187]. Moreover, DJ-1 levels in the CSF of sporadic PD patients were measured, and the results demonstrated that CSF DJ-1 levels were higher in PD patients than in healthy controls, and DJ-1 levels are higher in early-stage PD patients than in advanced-stage PD patients and healthy controls, which demonstrates the protective role of DJ-1 against oxidative stress in the early stages of the disease [188].

The serum immune marker profile is also informative of disease progression and hence can potentially be used as a prognostic biomarker. The first large-cohort study was carried out on newly diagnosed PD patients and control subjects to determine serum cytokine markers. According to the results, a panel of cytokines are closely associated with cognitive and motor features. TNFα, IL-1ß, IL-2, and IL-10 levels were higher in PD. The authors suggested that a higher pro-inflammatory profile was linked with decreased cognition and fast motor regression, whereas a lower pro-inflammatory profile was associated with enhanced cognition and retained motor functions [189]. Measured levels of IL-6 in serum from participants with PRKN/PINK1 mutations determined that individuals with biallelic PRKN/PINK1 mutations had higher IL-6 levels than healthy controls, lending support to the idea that inflammation plays a role in PRKN/PINK1-related PD. The results also demonstrated the potential of IL-6 as a progression marker in PD induced by mutations in PRKN/PINK1 [190]. The total number of T cells and subgroups of T helper cells (Th1, Th2, and Th17) from whole blood and serum inflammatory cytokine levels in the serum were examined. PD patients had a higher frequency of Th1 cells and IL-10 and IL-17A serum levels than controls. There was no significant association between microglial activation and peripheral inflammatory markers obtained from a multimodal PET study in the brains of PD patients [186] (Table 1).

### 4.2. Animal Studies

#### 4.2.1. Sporadic Models

Neurotoxins in animal models: neurotoxins, such as MPTP and 6-hydroxydopamine (6-OHDA) induce inflammatory responses leading to neurodegeneration. MPTP activates microglia and increases M1-associated pro-inflammatory cytokines, such as IL-6, IFNγ, and TNFα. According to reports, the glial response to MPTP peaks before dopaminergic neuron death [191,192]. In addition, it was reported that animals lacking IFNγ or TNFα are resistant to MPTP-induced neurodegeneration [149,193]. In another study, T cell (CD4+) infiltration into the SN was observed in MPTP-treated mice, and MPTP-induced dopaminergic neuron loss is reduced in T cell-deficient mice indicating a pro-inflammatory role of T cells (CD4+) in MPTP-induced neurotoxicity [47]. In other studies, microglial activation in the SN has been revealed by the dramatic amoeboid morphological change in the MPTP-induced PD model [191,194].

The 6-OHDA-induced PD model has also revealed a strong activation of microglia by immunohistochemistry and PET imaging [195]. In another rat model induced by 6-OHDA, early microglial activation was seen prior to the death of dopaminergic neurons and the observation of phagocytic microglia earlier than microglia that express the MHC II antigen [196]. These findings strongly suggest that microglia-mediated inflammation has a pathogenic role in PD.

LPS administration to rodent brains can induce certain sporadic PD symptoms, such as motor abnormalities and gradual degradation of the nigrostriatal dopaminergic system. LPS administration to the supranigral region of the rat brain induces rapid microglial activation followed by degeneration of nigrostriatal dopaminergic circuitry in a dose- and time-dependent manner [197,198]. Although the ventral tegmental region also contains dopaminergic neurons, direct injection of LPS causes dopaminergic cell death only in the SN. The greater percentage of microglia in the SN compared to other brain regions may be the cause of this particular neurotoxicity [199,200]. This leads to inflammatory processes that cause degeneration of dopaminergic neurons in the nigrostriatal pathway. Supporting this finding, other researchers studied the phenotypes of microglia in the nigrostriatal pathway by combining transcriptomics and in situ morphological approaches. These studies demonstrated that microglia have an inherent transcriptional immune-alerted signature that shares characteristics with inflammation-induced microglia and could have implications for PD [201].

In a different LPS-induced animal model, cell-to-cell transfer of human α-syn from the host striatum to naive neurons transplanted intrastriatally was quantified. Treatment with Plexxikon 5622 (PLX), a CSF1R kinase inhibitor, resulted in an 80% decrease in the number of microglia in the striatum. After PLX treatment, human α-syn immunoreactivity increased dramatically in the fraction of transplanted dopaminergic neurons demonstrating that microglia modulate the transmission of α-syn between neurons. These results imply that microglia participate in the cell-to-cell transmission of α-syn, which plays an important role in the PD mechanism [202]. Another study using the LPS-induced model of PD in rats showed an increase in pro-inflammatory cytokines during the first week of infusion. By the end of the second week, most oxidative stress markers were significantly increased. Finally, in the third week, apoptotic pathways were activated [64].

Prevention of the molecular pathways active in neurotoxic M1 phenotype in microglia reduced dopaminergic neuron loss in PD. For instance, Fasudil significantly reduced dopaminergic neuron loss, the accumulation of α-syn, and the inflammatory response with a transition from pro-inflammatory M1 microglia to anti-inflammatory M2 microglia. In the LPS-induced PD animal model, the transition from the M1 to M2 phenotype was accompanied by decreased NF-κB activation and IL-12 and TNFα formation in the SN and olfactory bulb. This research demonstrates the Fasudil’s function in preventing LPS-mediated dopaminergic neuron degradation and offers a therapeutic plan for the treatment of PD [203].

#### 4.2.2. Transgenic Models

Various transgenic animal studies demonstrated microglial activation in PD progression.

The α-syn model in a study using two CX3CR1 genotypes mice, *Cx3cr1*^+/+^ and *Cx3cr1*^−/−^, overexpressing human α-syn, both wildtype (α-syn^WT^) and mutant (α-syn^A53T^), showed that α-syn^A53T^-overexpressed *Cx3cr1*^–/–^ mice had high increase in neurodegeneration, neuroinflammation, and microglial activation [204]. In another study, Thy1-α-syn mice (line 61), transgenic mice overexpressing human wildtype α-syn [205], were used to assess microglial activation in different brain regions of mice of different ages. In mice with ages ranging from 1 to 14 months old, there was an obvious increase in levels of TNFα, MHC II, CD8, CD4, and TLRs. Microglial activation was detected at early months and persisted until the mice were 14 months old. Additionally, α-syn overexpression induced inflammatory response only in regions containing axons and cell bodies of the nigrostriatal neural pathway [206].

Activation of microglia in PD is not necessarily related with the death of dopaminergic neurons; however, in some protein-based transgenic models, such as double mutant α-syn^A30P^ and α-syn^A53T^ under TH promoter and α-syn^A53T^ under prion protein promoter (PrP), activation of microglia was observed early before dopaminergic neuron death [207,208]. This is also shown in a study using mice overexpressing human α-syn^WT^ under the TH promoter, where early microglial activation and TNFα elevation were demonstrated specifically in the SN of 1-month old mice [209]. Expression of human α-syn120, which is regulated by the TH promoter, in mice model shows aggregates of α-syn in the SN and changes in DA levels [210]. Expression of E46k human α-syn in mouse models showed α-syn inclusions also showed Tau inclusions in the SN [211]. These are examples showing that microglial activation depends on α-syn pathology and neurodegeneration and not on neuronal death.

It has been shown that α-syn overexpression by viral vectors causes early activation of microglia, and this might be influenced by neuronal death. This was shown by overexpression of α-syn by an adeno-associated virus (AAV) vector, which activated microglia early, produced inflammatory cytokines, and triggered an adaptive immune response without dopaminergic neuron loss [212].

Comparison between adenoviral (AV) overexpression of human α-syn in wild-type mice and Tlr2^−/−^-genotyped mice demonstrated that SNpc neurons that expressed α-syn in Tlr2^−/−^ mice significantly reduced microglial activation, and interestingly, TLR2 activation was associated with oligomeric α-syn rather than monomeric or fibrillar α-syn indicating that it was conformation dependent [57]. In another study that evaluates the function of TLR4 by creating a mouse model overexpressing α-syn and is deficient of TLR4. It was observed that the lack of TLR4 leads to increased α-syn aggregation and TNFα, loss of nigrostriatal dopaminergic neurons, and reduced microglial phagocytosis, which points out the importance of the role ofTLR4 in phagocytosis [213]. Likewise, only TLR4 deletion specifically reduces microglial activation and thus enhances the survival of dopaminergic neurons [214]. These findings indicate that animal models overexpressing either human wild-type or mutant α-syn display microglial activation and neuroinflammation.

The involvement of various PD-associated genes in the immune response of microglia and the release of pro-inflammatory cytokines related to it also have been studied via other transgenic animal models related to other causative genes in neuroinflammation-mediated PD.

Suppression of the PRKN gene in a murine model showed increased GSH levels, decreased astrocyte proliferation, and increased microglial proliferation [215]. Another study using a knockout mutation of PRKN and low LPS treatment for an extended period of time concluded that a deficiency of PRKN expression leads to a higher susceptibility to dopaminergic degeneration in the SN [216]. Moreover, the effect of aging in PRKN-knockout mice showed an abnormal increase in midbrain microglial activation [217]. The knockout mutation of LRRK2 in the rat model showed resistance to dopaminergic neuron degeneration, which is linked to decreased numbers of pro-inflammatory microglia in the SN [218]. PINK1-knockout mice brain slices confirmed elevation in the expression levels of pro-inflammatory cytokines, such as IL-1β, IL-6, and TNFα [219]. DJ-1^−/−^ mice treated with LPS showed a high expression of CD14, and reduced numbers of dopaminergic neurons were also detected in the SN by TH staining [220].

### 4.3. In Vitro Studies

Studies have revealed that extracellular α-syn causes microglia to respond in a pro-inflammatory manner by producing more inflammatory mediators, which have also been associated with microglia related cell toxicity [58]. However, it is suggested that fibrillar α-syn has a higher ability to cause an elevation in levels of these mediators [221].

One of the earliest reports of the ability of α-syn to induce a pro-inflammatory phenotype in microglia demonstrated that oligomeric α-syn was phagocytosed by microglia and that this resulted in PHOX NADPH activation leading to the ROS production and increased PGE2 [58]. Moreover, α-syn-induced microglia activation causes neuronal neurotoxicity through membrane translocation of NADPH subunits (p47 and p67) by ROS production and activation of PHOX NADPH oxidase. Furthermore, it was found that EP^−/−^ microglia exhibit decreased neurotoxicity due to the suppression of p47^phox^ translocation and increased degradation of α-syn [222]. Moreover, oligomeric α-syn triggers purinoceptor 7 (P2X7) receptor through PI3K/Akt activation and elevates oxidative stress by PHOX activation in BV2 microglial cells [223]. Microglial cells exposed to glimepiride, a sulphonylurea commonly used in diabetes treatment that causes the release of GPI-anchored proteins from cells, detached CD14 from the cell membrane and decreased the release of TNFα, IL-1, and IL-6 by microglial cells after exposure to α-syn. Additionally, glimepiride decreased α-syn-induced TLR4 translocation in membrane rafts [224]. Another study in BV2 cells treated with recombinant α-syn showed that TLR4 is mediated in the phagocytosis of α-syn via NF-κB nuclear translocation and causes the release of pro-inflammatory cytokines, such as IL-6, TNFα, and CXCL1. However, it has been shown that the absence of TLR4 does not completely abolish the inflammatory response in vitro suggesting the existence of parallel TLR4-independent mechanisms [213]. In addition, extracellular α-syn released from α-syn-overexpressed SH-SY5Y neuronal cells caused activation of microglia by binding to TLR2, and it is shown that oligomeric α-syn are the most potent agonists for TLR2 compared to monomeric and dimeric forms of α-syn [57]. All forms of α-syn affect microglial activation not only via TLRs but also via other proteins involved in activation, such as CD11b [225].

Isolated primary microglia treated with recombinant α-syn show that LRRK2-knockout microglia have a higher ability to clear α-syn explaining the negative regulation of LRRK2 of α-syn clearance [226]. Moreover, LRRK2 also has a positive regulation on the induction of inflammation, which was observed in an LRRK2-knockout BV2 microglial cell culture after treatment with LPS followed by a decrease in the levels of TNFα, iNOS, IL-1β, and IL-6 [227]. To see the role of PRKN in microglia, a study found that microglia treated with LPS had reduced Parkin mRNA and protein, which are believed to be inhibited by blocked NF-κB signaling [228]. In addition, deficiency of DJ-1 in microglia using short hairpin RNA (shRNA) resulted in high levels of cytokines, such as IL-6 and IL-1β, as well as intracellular ROS and NO, which show elevated dopaminergic neurotoxicity and low levels of anti-inflammatory TREM2 [229]. Additionally, DJ-1 deficiency showed poor extracellular α-syn uptake [230]. However, PINK1-mutated microglia cells stimulated by LPS and IFNγ reduced pro- and anti-inflammatory cytokines [231]. These results from in vitro studies clearly suggest that microglia play a significant role in the pathogenesis of PD.

## 5. Conclusions

The complicated etiology of PD is a drawback for designating efficient therapeutic strategies. As one of the important factors in the initiation and development of the disease, we have reviewed neuroinflammation in detail, especially focusing on the role of microglia as the main character of this process. Microglial activation and the features of their phenotypes are evaluated in the context of neuroinflammation in PD.

The interaction of microglia with other immune and non-immune cells, as well as with mediators of pro- and anti-inflammation, have been investigated in terms of the signaling pathways in which they are involved. This is important to emphasize as these interactions are important in the progression of neurodegenerative diseases, including PD.

The point of view we have covered in this review, microglia mediated neuroinflammation in PD, will be beneficial in designing new therapies taking not only the molecular targets of the disease but also the immune system into consideration.

## Figures and Tables

**Table 1:** Human studies as evidence for microglia-mediated neuroinflammation in PD. Increase (↑), decrease (↓).

Study	Participants	Target	Results
Postmortem Studies			
McGeer, P L et al. [75]	5 PD; 9 DAT; 2 ccerebrovascular accidents; 7 non-neurological controls.	Brain	↑ Number of Reactive Microglia in SNcp. ↑ MHC-II positive ameboid microglia.
Imamura, Kazuhiro et al. [178]	12 PD; 4 controls.	Brain	↑ MHC-II positive microglia in SN. ↑ MHC-II positive microglia in hippocampus, cingulate and temporal cortexes.
Croisier, Emilie et al. [179]	37 PD.	Brain	↓ Dopaminergic neuronal. ↑ α-syn in SN. ↑ CD-86+ phagocytes.
Reynolds, Ashley D et al. [180]	10 PD; 3 AD; 10 controls.	Brain	↑ NF-κB in SNpc in PD.
Smajić, Semra et al. [181]	5 idiopathic PD; 6 controls.	Brain	↑ ameboid microglia in SN.
Brochard, Vanessa et al. [47]	14 PD; 8 controls.	Brain	↑ CD4+ & CD8+ T cells in SN.
Brain PET Imaging			
Lavisse, Sonia et al. [185]	24 PD; 28 controls.	Brain	↑ reactivated microglia in midbrain.
Liu, Shu-Ying et al. [186]	24 early-stage PD; 23 controls.	Brain	↑ reactive microglia in putamen.
Extracellular Biological fluid studies			
Park, Min Jeong et al. [187]	23 naïve-drug PD; 28 neurological controls.	CSF	↑ oligomeric α-syn in PD.
Waragai, Masaaki et al. [188]	40 sporadic PD; 38 controls.	CSF	↑ DJ-1 in early-stage PD.
Williams-Gray, Caroline H et al. [189]	230 PD; 93 controls.	Serum cytokines	↑ TNFα, IL-1β, IL-2, and IL-10.
Borsche, Max et al. [190]	15 biallelic PRKN/PINK1 carrier; 19 affected heterozygous PRKN/PINK1 carrier; 15 unaffected heterozygous PRKN/PINK1 carrier; 59 idiopathic PD 90 controls.	Serum IL-6 and cell-free mtDNA	↑ IL-6 in biallelic PRKN/PINK1 mutations carriers. ↑ cell-free mtDNA in biallelic and heterogeneous PRKN/PINK1 mutation carriers.
Liu, Shu-Ying et al. [186]	24 early-stage PD; 23 controls.	Blood and serum cytokines	↑ Th1 cells in blood. ↑ IL-10 and IL-17A in serum.

Increase (↑), decrease (↓).

## Data Availability

Not applicable.
